# Characterization of Tissue Engineered Endothelial Cell Networks in Composite Collagen-Agarose Hydrogels

**DOI:** 10.3390/gels6030027

**Published:** 2020-09-03

**Authors:** Houda Ichanti, Sanja Sladic, Stefan Kalies, Axel Haverich, Birgit Andrée, Andres Hilfiker

**Affiliations:** 1 Leibniz Research Laboratories for Biotechnology and Artificial Organs (LEBAO), Department of Cardiothoracic, Transplantation and Vascular Surgery, Hannover Medical School, 30625 Hannover, Germany; Ichanti.Houda@mh-hannover.de (H.I.); Sladic.Sanja@mh-hannover.de (S.S.); Haverich.Axel@mh-hannover.de (A.H.); 2 Institute of Quantum Optics, Leibniz University Hannover, 30167 Hannover, Germany; kalies@iqo.uni-hannover.de; 3 Lower Saxony Centre for Biomedical Engineering, Implant Research and Development, 30625 Hannover, Germany

**Keywords:** collagen hydrogel, hybrid hydrogel, tissue engineering, endothelial cell network

## Abstract

Scaffolds constitute an important element in vascularized tissues and are therefore investigated for providing the desired mechanical stability and enabling vasculogenesis and angiogenesis. In this study, supplementation of hydrogels containing either Matrigel^TM^ and rat tail collagen I (Matrigel^TM^/rCOL) or human collagen (hCOL) with SeaPlaque^TM^ agarose were analyzed with regard to construct thickness and formation and characteristics of endothelial cell (EC) networks compared to constructs without agarose. Additionally, the effect of increased rCOL content in Matrigel^TM^/rCOL constructs was studied. An increase of rCOL content from 1 mg/mL to 3 mg/mL resulted in an increase of construct thickness by approximately 160%. The high rCOL content, however, impaired the formation of an EC network. The supplementation of Matrigel^TM^/rCOL with agarose increased the thickness of the hydrogel construct by approximately 100% while supporting the formation of a stable EC network. The use of hCOL/agarose composite hydrogels led to a slight increase in the thickness of the 3D hydrogel construct and supported the formation of a multi-layered EC network compared to control constructs. Our findings suggest that agarose/collagen-based composite hydrogels are promising candidates for tissue engineering of vascularized constructs as cell viability is maintained and the formation of a stable and multi-layered EC network is supported.

## 1. Introduction

Tissue engineering aims to develop in vitro tissues to improve or replace impaired biological tissues in vivo. To date, clinical applications of tissue engineering are limited to tissues which do not require vascularization nor anastomosis [1,2,3,4]. The 3D engineered tissues exceeding 200 µm (the effective diffusion limit of oxygen), which are densely populated with cells, quickly develop a necrotic core, if they are not vascularized [5]. Therefore, the vascularization of the engineered tissue is a major challenge in tissue engineering.

In the last decade, research advanced in pre-vascularization strategies with the aim to create vascular networks resembling the physiological capillary bed. The vascular network should be introduced into engineered tissues prior to implantation to allow microsurgical anastomosis to the host vasculature, followed by immediate perfusion of the engineered construct with blood. In general, vascularization strategies can be classified into three categories: Decellularization and re-endothelialization of vascularized tissues, micro-fabrication, and self-assembly.

In the case of decellularization, a preservation of organ/tissue vasculature is achieved through decellularization procedures by which cellular material is removed while maintaining the naturally existing 3D vasculature as well as the ECM structure. The maintained vascular tree is subsequently repopulated and re-endothelialized with human cells generating a perfusable construct while preventing thrombosis [6,7]. A major hurdle associated with the use of decellularized matrices of xenogeneic origin for vascularized tissue and transplantation in general, is the need of the complete removal of residual antigenic substances before widespread clinical application [8].

The micro-fabrication approach uses different technologies in an attempt to generate channel structures to guide endothelialization within 3D scaffolds [9]. Such structures are, however, still far from replicating the complex architecture and functionality of the capillary bed, making their immediate clinical translation challenging. Additionally, most micro-fabricated systems do not provide sites for direct anastomosis with the host vasculature and mainly rely on the rather slow process of ingrowth and subsequent connection to the host vasculature after subcutaneous transplantation, as a proof of concept for graft perfusion upon implantation [10,11,12].

The self-assembly strategy originates from the ability of ECs to inherently form vascular structures through a spontaneous assembly under pro-vasculogenic culture settings. This ability has been widely explored in both, in vitro and in vivo studies, with the goal to incorporate such vascular-like networks in engineered tissues. The self-assembly strategy for human vascularized tissues relies at least on three key elements: ECs forming a capillary-like vascular network, supporting cells stabilizing the capillary-like vascular network, and scaffolds allowing cell interactions and thus vascular-like network formation [13].

Scaffolds constitute an important element in vascularized tissues using self-assembly strategies. Different scaffolds have been investigated to provide the desired mechanical stability in 3D and to allow a vasculogenic and angiogenic remodeling [14]. For instance, in the context of bone tissue engineering, an attempt of generating vascularized bone construct was reported by Ying et al. where human blood-derived endothelial colony-forming cells (ECFCs) were encapsulated with bone marrow derived mesenchymal stem cells (MSCs) in a methacrylated gelatin hydrogel (GelMA) [15]. The MSCs induced the self-assembly of ECFCs into tubular structures with a lumen [15]. Additionally, the study demonstrated that MSCs provided coverage of the capillary like structures, expressing α-smooth muscle actin (α-SMA) and presenting pericyte-like phenotype [15]. In terms of scaffold’s mechanical properties, the self-assembly into vascular-like structures was highly dependent on the crosslinking extent and the stiffness of the GelMA, which could be easily tuned by polymerization with UV light, or the degree of methacrylation of the polymer [15]. For soft tissues, an engineered muscle flap for reconstruction of large soft tissue defects was reported by Shandalov et al. [16]. For that, a 3D biodegradable poly-l-lactic acid (PLLA)/poly(lactic-coglycolic acid) (PLGA) was used as scaffold to generate muscle tissue in vitro by a co-culture of myoblasts, fibroblasts, and endothelial cells [16]. The construct was cultured in vitro for 10 days until formation of small capillary net [16]. The tissue construct was then implanted in mice with the capillaries sprouted from the recipient’s femoral artery and vein [16]. The graft was finally transferred as a flap to fill a full-thickness abdominal wall defect [16]. The transferred flap showed full integration in the surrounding tissue and proved viable and well-vascularized while providing mechanical support to the abdominal wall [16]. Both natural and synthetic scaffolds are used for the formation of EC vascular-like network. Natural scaffolds comprise biopolymers such as collagens, fibrin, and gelatin matrices [17,18], while commonly used synthetic scaffolds include poly-l-lactic acid (PLLA) and poly-lactic-co-glycolic acid (PLGA) [19]. 

Previously, our group has used natural scaffolds either as an animal-derived composite of Matrigel^TM^/rCOL or xeno-free hCOL only [20,21]. The combination of human umbilical vein endothelial cells (HUVECs) and human adipose tissue-derived stromal cells (hASCs) in a hydrogel construct containing hCOL cultured in serum-free medium supported the formation of stable EC network; however, it led to thinner and a single-layered EC network compared to a multi-layered network in Matrigel^TM^/rCOL [21]. Here, we investigate the use of agarose in combination with collagen as composite hydrogel scaffolds to support the formation of 3D EC networks.

Agarose is a polysaccharide of 3,6-anhydrous-L-galactose and D-galactose repeating units [22]. Upon gelation, agarose forms a rigid and porous network and it is often investigated in the context of therapies for central nervous system injury and disease, where it supports neurite extension [23]. Agarose can form interpenetrating networks with some natural hydrogels, such as collagen, chitosan, and gelatin [24,25,26]. It also allows biomolecules such as RGD to be covalently linked to agarose’s polysaccharide chains. In addition, biomolecules can be also physically entrapped in the agarose. Immobilizing biomolecules in agarose hydrogel improves its bio-compatibility and bio-activity [27]. In the context of vascularized tissue engineering, collagen-agarose composite hydrogels offer the possibility to 3D print collagen material, which is otherwise difficult to achieve using native collagen only [28].

In this study, SeaPlaque^TM^ agarose was used. It is a low temperature melting agarose with a gelling temperature between 20 °C and 30 °C. The effect of hydrogel composition on the thickness of the construct and the EC network structure was investigated.

## 2. Results and Discussion

### 2.1. High Concentration of rCOL Increases Construct Thickness but Does Not Support the Formation of an EC Network 

A modification of the gel composition could lead to an increase in construct thickness by tuning the gel stiffness and consequently reducing gel shrinkage. This can be achieved by increasing the protein concentration while leaving the cell density unchanged. To test this hypothesis, the concentration of rCOL was increased from the standard content of 1 mg/mL to 3 mg/mL while maintaining the Matrigel^TM^ concentration (0.9 mg/mL) in Matrigel^TM^/rCOL constructs.

**Figure 1:**
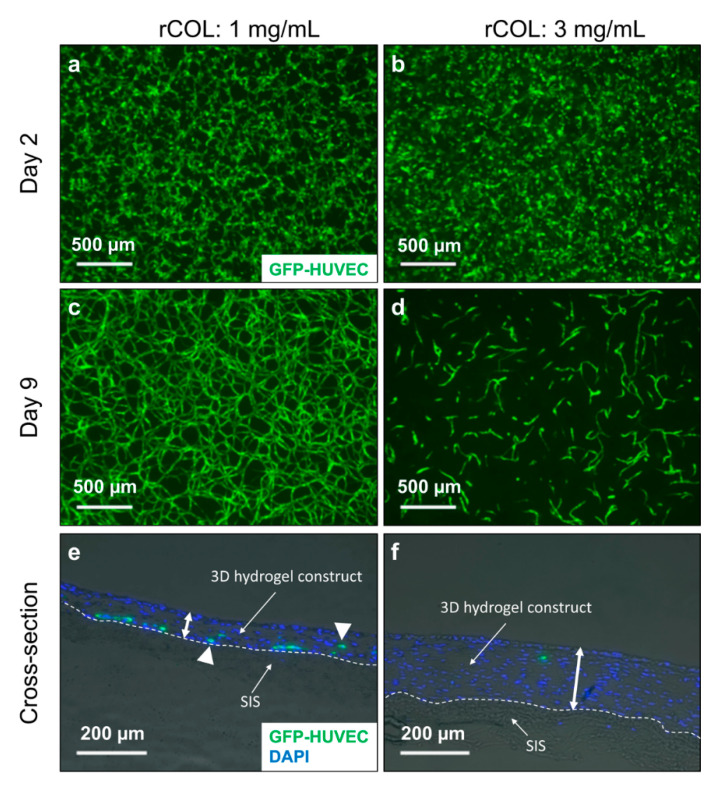
Assessment of GFP-HUVEC self-assembly into a network when co-cultured with unlabeled hASCs in 3D hydrogels of different rCOL concentrations. (**a**,**c**) Top view of 3D construct showing GFP- HUVEC in a hydrogel containing Matrigel^TM^ and 1 mg/mL rCOL after 2 and 9 days in culture, respectively. (**b**,**d**) Top view of 3D construct showing GFP-labeled human umbilical vein endothelial cells (HUVEC) in a hydrogel containing Matrigel^TM^ and 3 mg/mL rCOL after 2 and 9 days in culture, respectively. (**e**,**f**) 4’,6-diamidino-2-phenylindole (DAPI) staining of cross sections of GFP-HUVEC/hASC constructs containing Matrigel^TM^ and 1 mg/mL or 3 mg/mL rCOL, respectively. White double arrows depict the height of the 3D hydrogel construct, the dashed lines depict the border between SIS and 3D hydrogel construct, and white triangles exemplary depict GFP-HUVECs. Scale bar: (**a**–**d**): 500 µm, (**e**,**f**): 200 µm.

After 2 days of culture, first signs of green fluorescent protein labeled-HUVEC (GFP-HUVEC) self-assembly were observed in the standard gel containing 1 mg/mL rCOL (Figure 1a). In contrary, GFP-HUVECs exhibited a round morphology in 3 mg/mL rCOL (Figure 1b). After 9 days of culture, a formation of a stable HUVEC network was observed in control construct (Figure 1c), while a low GFP-HUVECs cell number was observed in 3 mg/mL rCOL hydrogels developing a more elongated morphology compared to day 2 and forming only short vascular-like structures (Figure 1d). 

This increase in rCOL concentration resulted in a drastic increase of construct thickness in the 3 mg/mL rCOL constructs (~200 µm) compared to the control containing 1 mg/mL rCOL (~60 µm), as assessed through analysis of DAPI staining of cross-sections after 2 weeks in culture (Figure 1e,f). However, the 3 mg/mL rCOL containing hydrogel did not support the spontaneous formation of an EC network. This is probably due to the increase of scaffold’s stiffness as previously suggested by Rao et al. [29]. In their study, Rao and colleagues investigated the degree of vascular-like network formation by HUVECs when co-cultured with MSCs in 3D collagen/fibrin gel [29]. An increase of collagen I content resulted in cell death and thus a decreased HUVEC network formation [29]. Measuring the mechanical properties of the matrices showed that the latter increased with increasing collagen I content. This indicates that increasing mechanical stiffness of the matrix correlates inversely with EC vascular-network formation [29]. Ideally, the self-assembled vascular-like network generated in our study is intended to be used for the pre-vascularization of various engineered tissues including those exhibiting high mechanical properties. In a such case, the use of composite matrices was reported to successfully support the formation of EC vascular-like networks. For instance, the addition of nanoparticulate hydroxyapatite, bone-like mineral, to collagen I/fibrin matrices was shown to reduce the matrix compaction while maintaining high mechanical properties. The formation of a HUVEC vascular-like network was observed in co-culture with human bone marrow-derived MSCs in such a material [30]. In a different study, attempts were made to improve the mechanical stability of scaffolds for vascularization by self-assembly by using a photopolymerizable gelatin methacrylate (GelMA) hydrogel [15]. ECFCs and MSCs in the GelMA hydrogels generated extensive capillary-like networks in vitro [15]. The capillary-like network established a functional anastomosis with host vasculature, when subcutaneously implanted into immunodeficient mice [15].

In our study, the increase of rCOL in Matrigel^TM^/rCOL did not elicit the desired response. Therefore, another approach was followed by supplementing the original construct composition with agarose.

### 2.2. Agarose-Supplementation Supports 3D EC Network Formation in Matrigel^TM^/rCOL and hCOL 

**Figure 2:**
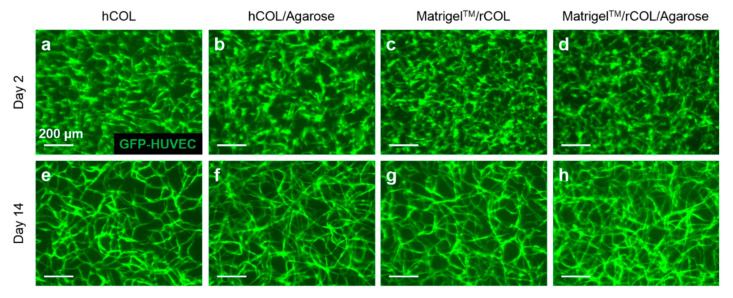
HUVECs self-assemble into an EC network when co-cultured with unlabeled-hASC in 3D hydrogel containing 0.03% SeaPlaque^TM^ agarose. 3D hydrogel constructs containing GFP-HUVECs after 2 (**a**–**d**) and 14 days (**e**–**h**) of culture using hCOL (**a**,**e**), hCOL/Agarose (**b**,**f**), Matrigel^TM^/rCOL (**c**,**g**), Matrigel^TM^/rCOL/Agarose (**d**,**h**). EC network formation started at day 2 of cultivation in all hydrogel settings. The formed networks were stable during the cultivation period of 14 days. Scale bar: 200 µm.

SeaPlaque^TM^ agarose was added to the hydrogel containing cells and Matrigel^TM^/rCOL or hCOL at a final concentration of 0.03%. At day 2 of culture, no difference in cell density was observed between the control constructs containing hCOL or Matrigel^TM^/rCOL (Figure 2a,c) and constructs supplemented with agarose (Figure 2b,d). Under prolonged culture for 14 days, the GFP-HUVECs self-assembled into a vascular-like network in both control constructs (Figure 2e,g) and those supplemented with agarose (Figure 2f,h). In addition, from visual inspection of the 2D fluorescence images, the networks formed in agarose-containing constructs seemed to be denser (Figure 2f,h) than those formed in controls (Figure 2e,g). Additionally, unlike a high concentration of rCOL, the use of SeaPlaque^TM^ agarose did not cause tremendous cell loss. This finding is in line with a study, where Kreimendahl and colleagues used a 3D printable agarose-type I collagen blend and enhanced the formation of capillary-like structures by HUVECs when co-cultured with human dermal fibroblasts for 14 days in DMEM [28]. The study has assessed the formation of HUVEC capillary-like structures upon modification of agarose content and showed that a content of 0.5% resulted in a minimal cell loss while enabling the formation of HUVEC networks. A higher agarose content led to cell loss and therefore failed to support the formation of HUVEC networks. This might be attributed to the agarose-induced disruption of collagen remodeling by cells as demonstrated in a separate study [31]. Ulrich et al. showed that agarose strongly impairs cell-directed assembly of large collagen bundles by structurally coupling and reinforcing the collagen fibers which results in steric barriers to cell motility [26,31]. In our study, a concentration of 0.03% agarose was investigated as a proof of concept; higher agarose concentrations could be tested for future analysis to investigate the extent to which EC networks can form at higher agarose concentrations.

### 2.3. The Supplementation of Matrigel^TM^/rCOL Construct with SeaPlaque^TM^ Agarose Leads to Thicker Hydrogel Constructs

**Figure 3:**
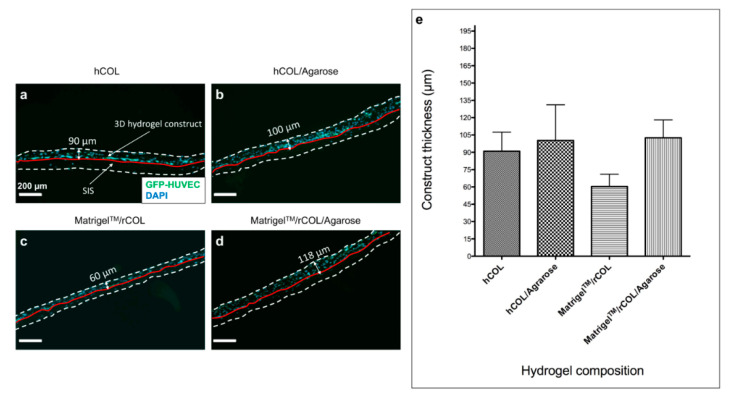
Construct thickness increased slightly in agarose supplemented hCOL and considerably in Matrigel^TM^/rCOL constructs. (**a**–**d**) Images of cryo-sections from 3D hydrogel constructs in hCOL (**a**), hCOL/Agarose (**b**), Matrigel^TM^/rCOL (**c**), Matrigel^TM^/rCOL/Agarose (**d**). The white dashed lines define the limits of the hydrogel and SIS construct while the red solid line indicates the hydrogel/SIS border. The hydrogel construct is located on the upper side of the red line and contains HUVECs and hASCs visualized with DAPI staining (**a**–**d**). The up down arrows indicate the average construct thickness for each hydrogel setting with 90 µm for hCOL (**a**), 100 µm for hCOL/Agarose (**b**), 60 µm for Matrigel^TM^/rCOL (**c**), and 118 µm for Matrigel^TM^/rCOL/Agarose (**d**). (**e**) A calculation of construct thickness was done by imaging of three different cryosections for each hydrogel setting (one construct analyzed per setting). The cross-section of the whole construct was imaged (approximately 9 images per cryosection) and five random thickness measurements were taken per image. Hydrogel thickness is presented as mean ± standard deviation (SD). Scale bar: 200 µm.

The construct thickness was measured based on imaging of three cryosections for one analyzed construct per setting. Images of the whole cryosection were taken (Figure 3a–d) and five random thickness measurements were taken per image (roughly 8–9 images per cryosection). The mean thickness was calculated from 128-132 measurements per hydrogel setting.

The average thickness of hCOL-constructs (90 µm) is slightly lower than in hCOL/Agarose construct (100 µm) showing that agarose supplementation did not change the thickness of the hydrogel in hCOL-based constructs (Figure 3a,b,e). Interestingly, the addition of agarose to the hydrogel containing Matrigel^TM^/rCOL contributed to a drastic increase in construct thickness of 100% from 60 µm in control constructs (Figure 3c,e) to approximately 118 µm in agarose containing constructs (Figure 3d,e). The thickness of agarose-free constructs in Matrigel^TM^/rCOL and hCOL is different even though both constructs contain the same total protein content. However, different protein compositions with hCOL made of over 97% Collagen I, while Matrigel^TM^/rCOL is composed of rat collagen type I and a mixture of basement membrane proteins (Matrigel^TM^)—mainly laminin, collagen IV, entactin [32]—results in a higher collagen I content in hCOL constructs compared to Matrigel^TM^/rCOL hydrogels. It is reported that endothelial cells mediate collagen I contractions, which are inversely related to collagen concentration [33]. Additionally, the hCOL and rCOL are derived differently from different sources. hCOL is derived from extracellular matrix secreted by human fibroblasts while rCOL is produced by the digestion of rat tail tissue. The different extraction methods of collagen may lead to different collagen fibers properties (e.g., size, length, and molecular weight) and thus collagen monomers and/or oligomers with different properties. Additionally, it was demonstrated that type I collagen oligomers affect in-vitro polymerization in terms of kinetics, fibril microstructure, and final mechanical properties of formed matrices [34,35]. This may lead to variations in remodeling of the hydrogel by cells and its contraction resulting in different hydrogel thicknesses [36]. This can explain the lower construct thickness in Matrigel^TM^/rCOL compared to hCOL. As for the increase of thickness in agarose containing constructs, this can be attributed to an increase of hydrogel density by solidified agarose, which is a very slow degrading material and is indigestible for mammalian cells. 

### 2.4. Supplementation of hCOL Construct with SeaPlaque^TM^ Agarose Leads to Thinner Cords but a Multi-Layered EC Network

**Figure 4:**
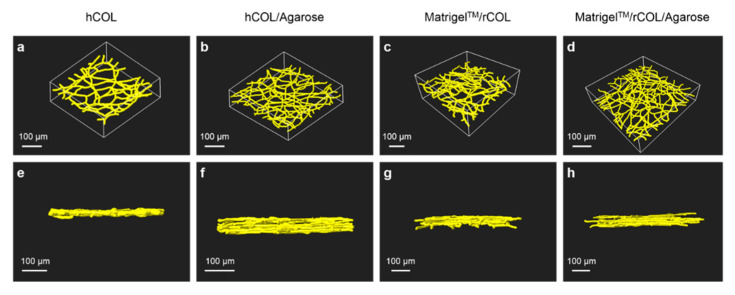
A 3D reconstruction of EC networks revealing the effect of agarose supplementation on the network structure and thickness. 3D reconstruction of multiphoton images obtained from constructs containing GFP-HUVECs and hASCs in hCOL (**a**,**e**), hCOL/Agarose (**b**,**f**), Matrigel^TM^/rCOL (**c**,**g**) or Matrigel^TM^/rCOL/Agarose (**d**,**h**) cultivated for 14 days. (**a**–**d**) 3D reconstruction of EC network employing the calculated mean diameter for each hydrogel setting. (**e**–**h**) Side view of the 3D reconstruction demonstrating the height of the EC network. Networks generated in agarose supplemented hCOL hydrogel are multi-layered with larger height and exhibit a smaller mean diameter compared to constructs generated with hCOL which have a mono-layer structure with thinner height. Networks generated in constructs generated with Matrigel^TM^/rCOL/Agarose are multi-layered with same height and exhibit a slightly smaller mean diameter compared to networks in control hydrogel containing Matrigel^TM^/rCOL hydrogel. Scale bars: 100 µm.

The quantification of the network parameters in constructs containing either hCOL/Agarose or Matrigel^TM^/rCOL/Agarose was performed on the basis of 3D reconstruction of the network using z-stacks of the EC network obtained by multiphoton microscopy which were processed using Imaris software (Figure 4, Table 1).

A comparison of the reconstructed EC networks in the different hydrogel compositions showed a more filigree network with smaller mean diameter in construct containing hCOL/agarose (Figure 4b) compared to network formed in agarose-free hCOL construct (Figure 4a). Additionally, the network in hCOL seemed to resemble a monolayer and has a smaller height (Figure 4e) compared to a multi-layered network in hCOL-Agarose construct (Figure 4f). These differences are confirmed by quantification of network parameters in constructs containing hCOL/Agarose. The latter has a smaller network mean diameter (7.7 ± 2.6 µm, Table 1) compared to agarose-free hCOL as shown in a previous study by our group (12.2 ± 3.3 µm, Table 2 [21]). A larger mean diameter in hCOL contributes to a larger network volume ((10.4 ± 0.8) × 10^5^ µm^3^, Table 2 [21],) compared to hCOL/Agarose construct ((6.9 ± 0.9) × 10^5^ µm^3^, Table 1). Additionally, other network parameters (number of nodes, total branching length, and number of segments) are larger in hCOL/Agarose constructs compare to agarose-free hCOL, which explains the more filigree structure of the network in hCOL/Agarose (Figure 4a,b, Table 1, Table 2 [21]). As for the Matrigel^TM^/rCOL setting, a slightly larger network diameter was observed in the control construct containing Matrigel^TM^/rCOL (Figure 4c) compared to the Matrigel^TM^/rCOL/Agarose construct (Figure 4d). This was further confirmed through network quantification with a mean diameter of 7.7 ± 2.6 µm (Table 1) for network containing Matrigel^TM^/rCOL/Agarose compared to 9.3 ± 2.4 µm for Matrigel^TM^/rCOL as shown in a different study by our group [21]. Except number of segments, other network parameters showed no significant differences (Table 1, Table 2 [21]) explaining the similar network structure in both agarose-containing and agarose-free Matrigel^TM^/rCOL constructs. The aim of generating such constructs with stable EC network is to provide a platform for clinically applicable prevascularized scaffolds with capillary-like functionality. The EC networks formed in agarose-collagen composite hydrogel have an average diameter range (7.2–7.7 µm) close to the size of large capillaries [37] and therefore have the geometrical potential to supply cells within an engineered tissue if connected to a perfusion system. 

The difference in network height observed in hCOL-based constructs can be attributed to either cell loss taking place in hydrogel constructs containing hCOL or cell division (not addressed in this study) occurring in hydrogel constructs containing hCOL/agarose. In the context of angiogenesis, it has been reported that the VEGF-mediated endothelial cell proliferation can be associated with increased matrix stiffness [38]. Therefore, future investigation should address the effect of agarose supplementation on the mechanical properties of collagen-agarose composite hydrogels and on cell fate over cultivation time. 

### 2.5. hASCs/HUVECs Interaction in Agarose-Containing Constructs

**Figure 5:**
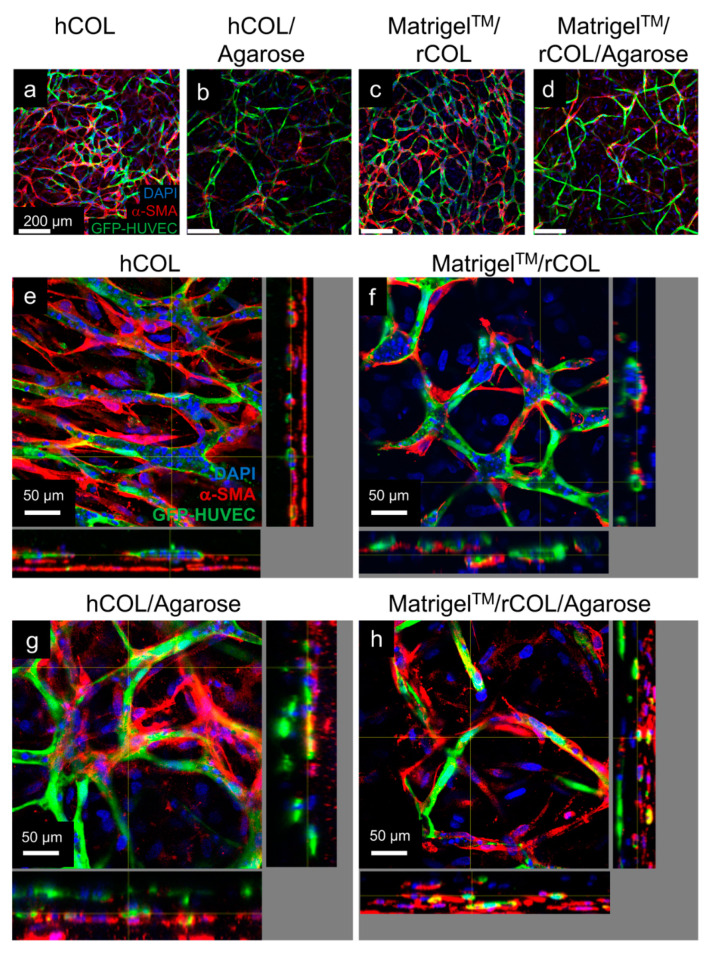
Confocal images of GFP-HUVECs co-cultured with hASCs and stained for α-SMA. Constructs containing hCOL (**a**,**e**), hCOL/Agarose (**b**,**g**), Matrigel^TM^/rCOL (**c**,**f**), and Matrigel^TM^/rCOL/Agarose (**d**,**h**) were analyzed after 14 days of cultivation. (**a**–**d**) Confocal images displaying an overview in a low magnification. (**e**–**h**) Virtual stacks of high magnification images with respective orthogonal views. α-SMA positive cells wrap around GFP-HUVEC cords under all conditions. Scale bar: (**a**–**d**): 200 µm, (**e**–**h**): 50 µm.

To assess the interaction of hASCs and HUVECs in agarose-containing constructs, staining against alpha-smooth muscle actin of whole constructs was performed at the end of the cultivation period of 14 days. Confocal microscopy analysis of the respective controls (Figure 5a,e for hCOL, Figure 5c,f for Matrigel^TM^/rCOL) and the agarose supplemented constructs showed α-SMA positive cells surrounding the cords of GFP-labelled HUVECs in both constructs, hCOL/agarose (Figure 5b,g) and Matrigel^TM^/rCOL/agarose (Figure 5d,h), which indicates that agarose does not prevent direct contact of hASCs with HUVECs, but instead acts as a scaffold by providing the physical space for HUVECs and hASCs to interact and finally develop mature vascular-like networks. Similar findings are reported in studies using collagen either alone or with fibrin as composite hydrogels. For the latter, Peterson et al. showed that collagen-fibrin matrix efficiently entrapped human ECs and fibroblasts in a 3D co-culture setting by supporting their viability and spreading [39]. ECs formed networks that were visible at day 7 of culture and more prominent by day 14 of culture with fibroblasts co-localizing with ECs. Additionally, in our study, orthogonal views of the confocal images show no obvious cord diameter differences between constructs containing Matrigel^TM^/rCOL (Figure 5f) and those containing Matrigel^TM^/rCOL/Agarose (Figure 5h). In contrary, larger cords are observed in hCOL hydrogel constructs (Figure 5e) compared to hCOL/Agarose constructs (Figure 5g). The reason for this difference is unclear, but it can be hypothesized that an increase of hydrogel stiffness, by addition of agarose, can lead to decreased cord diameter in EC networks. A similar pattern was reported in a study by Whisler et al., where fibrin was used at different concentration for the co-culture of HUVECs and human lung fibroblasts [40]. A higher concentration of fibrin resulted in a decreased cord diameter in the EC network. It is noteworthy that a strong increase in hydrogel stiffness may alter the formation of EC networks as reported elsewhere [29]. Therefore, an optimal value of hydrogel stiffness for the respective set-up needs to be determined. In addition, confocal images underlined the distribution of the EC network in agarose supplemented constructs throughout a larger height (Figure 5g,h) than constructs without agarose (Figure 5e,f). Furthermore, the multi-layered structure of the network is apparent in agarose supplemented hydrogels (Figure 5g,h). Therefore, the EC network in the overview pictures (Figure 5b,d) looks less dense compared to the respective controls (Figure 5a,c), as just one focal plane is depicted.

## 3. Conclusions

In this study addition of SeaPlaque^TM^ agarose to our standard Matrigel^TM^/rCOL hydrogel has noticeably contributed to an increase of the hydrogel thickness while preserving the formation of HUVEC vascular-like networks. In the case of hCOL, the agarose supplementation did not reveal differences in terms of construct thickness. Importantly, however, agarose has led to the formation of a multi-layered EC network compared to control constructs containing hCOL only while a multi-layered EC network was achieved in Matrigel^TM^/rCOL-based hydrogel with or without agarose. Further work should focus on assessing the effect of agarose on the biomechanical properties of the agarose-collagen composite hydrogels. Several agarose-based products have been FDA approved and are in use clinically as dermal fillers without adverse effects [41,42,43]. Additionally, owing to the agarose’s reversible thermos-gelling behavior and non-cytotoxic effects, agarose-based hydrogels can be exploited to develop a wide range of biomaterials for tissue engineering applications. 

Finally, a main challenge of pre-vascularized scaffolds is the establishment of a connection to perfusion system in vitro and to a suitable system in the host circulatory system in vivo. 

## 4. Materials and Methods 

### 4.1. Ethics Statement

Patient material was processed following approval of the Ethics Committee at Hannover Medical School (file reference 3475-2017) and after obtaining written informed consent from the patients. All tissues were used anonymously for this study. All experiments were performed in accordance with relevant guidelines and regulations.

### 4.2. Preparation of Small Intestinal Submucosa (SIS)

The preparation of decellularized small intestinal submucosa (SIS) was performed as previously described [44]. In brief, porcine small intestinal segments were isolated from German landrace pigs (18–25 kg) and stored in undiluted Braunol (7.5% povidone-iodine solution in water, B. Braun, Melsungen, Germany) at 4 °C. *Tunica mucosa* and *tunica serosa* of intestinal segments were mechanically removed, followed by a chemical decellularization in 1% Triton X-100 in 10 mM TRIS, pH 7.5 under continuous shaking (90 rpm) at room temperature for 24 h. Afterwards, SIS was washed with distilled water for 24 h under continuous shaking, followed by washing with phosphate buffered saline (PBS) supplemented with 1 g/L Vancomycin, 100 mg/L Gentamicin, and 2.5 mg/L Amphotericin B under continuous shaking for 10 days at room temperature with daily change of washing buffer. Three different drugs were added to the washing solution to prevent the growth of gram-positive bacteria (Vancomycin), gram-negative bacteria (Gentamicin), and fungi (Amphotericin B). Finally, SIS in a small amount of PBS was sterilized by 150 Gy gamma-ray irradiation (Gamma cell, Mölsgaard Medical Denmark, Kopenhagen, Denmark) and stored in PBS at 4 °C until further use for a maximum of 6 months. Before use, SIS was cut open along the longitudinal axis, fixed in a metal frame with the submucosal side facing up and covered with culture medium.

### 4.3. Human Umbilical Vein Endothelial Cells (HUVECs)

HUVECs obtained from pooled donors were purchased from Lonza. Cells were cultured in Endothelial Growth Medium 2 (EGM-2, Lonza, Basel, Switzerland). Passages 5–7 were used for all experiments.

### 4.4. Isolation of Adult Human Adipose Tissue-Derived Stromal Cells (hASCs)

Human adult adipose tissue-derived stromal cells (hASCs) were isolated from human tissues that were obtained from patients undergoing abdominoplasty. Isolation was performed according to a previously published protocol with minor modifications [45]. In brief, fat and connective tissue were minced, followed by addition of 10 mL Collagenase type II solution (760 U/mL, Worthington, Lakewood, NJ, USA) per 20 mL of tissue and incubation for 1 h at 37 °C under shaking. Fatty supernatants were collected and washed with PBS. After centrifugation, the cell pellet was washed with PBS, resuspended and cells were cultivated in EGM-2. The medium was changed after 48 h followed by a medium change every other day. Cells from passages 2–4 were used for the experiment.

### 4.5. Preparation of Lentiviral Supernatants and Cell Transduction

Lentiviral transductions of HUVECs were carried out under biosafety level 2 (S2) regulations. For the transduction, HUVECs in an early passage (P1 or P2) were seeded into T75 cell culture flasks in EGM-2. The medium containing the virus was replaced after 48 h. Efficiency of transduction was monitored microscopically by estimating the proportion of GFP positive cells or by FACS analysis [46].

### 4.6. Preparation of 0.4% SeaPlaqueTM Agarose

For the preparation of 100 mL of 0.4% SeaPlaque^TM^ agarose (Lonza, Basel, Switzerland), 0.8 g of SeaPlaque^TM^ agarose powder was added to an Erlenmeyer glass flask containing 100 mL of Ampuwa water. The mixture was boiled until the agarose was completely dissolved. The solution was further diluted 1:1 in serum-free medium [21] to prepare a 0.4% agarose solution.

### 4.7. Generation of 3D Hydrogel Constructs in Serum-Free Culture

For the generation of 3D hydrogel constructs containing GFP-HUVECs and hASCs in rCOL and Matrigel^TM^ cultured in serum-free medium, slight modification of previously published protocol from our group was used [20] (Tables S1–S5). Briefly, rCOL (Trevigen Inc., Gaithersburg, MD, USA) or hCOL (Sigma-Aldrich, Taufkirchen, Germany), double distilled water (ddH_2_O), gel medium, and 0.4 mM NaOH were mixed. For constructs containing Matrigel^TM^, 31 µL of Matrigel^TM^ (BD Biosciences, Billerica, MA, USA) was added. A mixture of 1.7 × 10^6^ GFP-HUVECs and 3 × 10^6^ hASCs was resuspended in serum-free medium and mixed with the hydrogel. For constructs supplemented with 0.4% SeaPlaque^TM^ agarose, 57 µL is added to the hydrogel mixture which was cast onto SIS-based casting mold. After 1 hour of incubation in a humidified incubator at 37 °C and an atmosphere with 5% CO2, solidified hydrogel constructs were covered with serum-free medium. The medium change was done every 2 days and the ECs network assembling was documented using a Stereo Discovery.V8 microscope equipped with an AxioCam camera and an HXP lamp (Carl Zeiss, Oberkochen, Germany.

### 4.8. Assessment of hASCs/HUVECs Interaction Using Immunofluorescence Staining

The staining procedure was performed according to a standard protocol and is described below. At the end of the experiment, whole 3D constructs were fixed with 4% PFA at RT for 30 min followed by 3 washing steps, 5 min each, using PBS. Constructs were placed on glass slides. The construct specimens were permeabilized with 0.3% Triton X-100 in PBS for 1 hour at room temperature and blocked with 2% donkey serum in PBS for 40 min at RT followed by overnight incubation with anti-human α-smooth muscle actin (α-SMA) (M0851, 1:400, DAKO, Santa Clara, CA, USA) at 4 °C. Specimens were washed three times for 5 min each with PBS followed by incubation with Cy3-labelled donkey anti-mouse antibody (1:300, Jackson ImmunoResearch, West Grove, PA, USA) for 2 h at RT. Specimens were washed three times with PBS for 5 min each. Nuclei were counterstained with DAPI (1 µg/mL in PBS) for 15 min at RT. After three additional washing steps with PBS for 5 min each, constructs were covered with fluorescent mounting medium and glass coverslips. The specimens were left to dry for at least 24 h at 4 °C. Specimens were analyzed using an Olympus FluoView 1000 confocal laser scanning microscope (Olympus Europe, Hamburg, Germany). 

### 4.9. Assessment of Constructs Thickness 

At the end of the experiment, the whole 3D hydrogel constructs were fixed with 4% PFA at RT for 30 min to preserve the construct structure and the GFP signal for subsequent fluorescence microscopy. After fixation, the constructs were washed three times with PBS and stored at 4 °C until further analysis. For cryosection preparation constructs were transferred into optimal cutting temperature compound (OCT) and left for 24 h at 4 °C. The OCT-embedded constructs were frozen in liquid nitrogen and stored at −80 °C. The frozen constructs were mounted on a cryostat and 5 µm cryosections were prepared and transferred onto superfrost glass slides. The cryosections were dried at RT for 2 h before storage at −80 °C.

Prior to nuclear staining using DAPI, the specimens were encircled with a PAP Pen and washed three times with PBS for 5 min each to remove residuals of the water-soluble OCT. Nuclei were stained with DAPI (1 µg/mL in PBS) for 15 min at RT. After three washing steps with PBS for 5 min each, the stained cryosections were covered with fluorescent mounting medium and glass coverslips. The specimens were left to dry for at least 24 h at 4 °C before they were analyzed using an Oberserver. A1 microscope. An estimation of construct thickness was done by imaging of 3 different cryosections for each hydrogel setting (one construct analyzed per setting). An imaging of the whole cryosection was done (approximately nine images per cryosection) and five random thickness measurements were taken per image. Hydrogel thickness is presented as mean ± SD.

### 4.10. 3D Reconstruction and Quantification of the EC Network

Image acquisition of the EC network formed by GFP-HUVECs was performed using a multiphoton microscopy setup employing a Chameleon Ultra II laser system running at 790 nm or 850 nm (Coherent Inc., Santa Clara, CA, USA), a Thorlabs MPM 200 multiphoton microscope body (Thorlabs, Newton, NJ, USA), and an Olympus XLPlan N objective (25×, NA 1.05). Z-stacks were taken at three random regions of each hydrogel setting (one construct analyzed per setting) with a z-step size of 1 μm. The 3D reconstruction of the EC network was performed with Imaris software (Bitplane, Zürich, Switzerland) using multiphoton z-stacks. The 3D quantification of EC network parameters was performed employing an AutoPath algorithm of FilamentTracer in the Imaris software. Data are given as mean ± SD for mean diameter, number of nodes, total branching length (sum of the lengths of all segments within the 3D network), network area (the sum of areas of all segments within the 3D network), network volume (the sum of volumes of all segments within the 3D network), and number of segments. Data were analyzed with unpaired *t*-test. A *p*-value ≤0.05 was determined as significant. 

## Figures and Tables

**Table 1:** Comparison of network parameters in agarose supplemented constructs. Constructs containing GFP-HUVECs and hASCs in hCOL/Agarose and Matrigel^TM^/rCOL/Agarose were cultured for 14 days. After cultivation period, z-stacks of constructs were taken at three random regions per construct using multiphoton microscopy. The stacks were processed with Imaris for 3D reconstruction and network quantification. Network parameters are presented as mean ± SD for: mean diameter, number of nodes, total branching length, network area, network volume, and number of segments. Data were analyzed with unpaired *t*-test. (*) *p*-value ≤ 0.05, ns: not significant.

	Matrigel^TM^/rCOL/Agarose	hCOL/Agarose	Significance
Mean Diameter (µm)	7.2 ± 2.2	7.7 ± 2.6	ns
Number of nodes	137.6 ± 20.2	102.0 ± 11.2	ns
Total branching length (µm)	15,300.0 ± 1248.9	13,366.6 ± 1222.0	ns
Network area (µm^2^)	(3.4 ± 0.45) × 10^5^	(3.2 ± 0.2) × 10^5^	ns
Network Volume (µm^3^)	(6.7 ± 1.2) × 10^5^	(6.9 ± 0.9) × 10^5^	ns
Number of segments	250.3 ± 34.2	182.3 ± 17.0	*

**Table 2:** Constructs containing GFP-HUVECs and hASCs in Matrigel^TM/^rCOL and hCOL were cultured for 14 days. After cultivation period, z-stacks of constructs were taken at three random regions per construct using multiphoton microscopy. The stacks were processed with Imaris for 3D reconstruction and network quantification. Network parameters are presented as mean ± SD for: mean diameter, number of nodes, total branching length, network area, network volume, and number of segments. Data were analyzed with unpaired *t*-test. (*) *p*-value ≤ 0.05, ns: not significant. Data re-used from Andrée et. al. [21].

	Matrigel^TM^/rCOL	hCOL	Significance
Mean Diameter (µm)	9.3 ± 2.4	12.2 ± 3.3	ns
Number of nodes	113.0 ± 19.3	81.7 ± 4.0	ns
Total branching length (µm)	10,747.0 ± 938.9	8357.3 ± 526.6	*
Network area (µm^2^)	(3.2 ± 0.3) × 10^5^	(3.2 ± 0.1) × 10^5^	ns
Network Volume (µm^3^)	(7.7 ± 1.0) × 10^5^	(10.4 ± 0.8) × 10^5^	*
Number of segments	207.3 ± 34.6	146.3 ± 4.2	*

## Supplementary Materials

The following are available online at https://www.mdpi.com/2310-2861/6/3/27/s1, Table S1: Control hydrogel construct containing Matrigel^TM^/rCOL in serum-free medium, Table S2: Control hydrogel construct containing hCOL in serum-free medium, Table S3: Hydrogel construct containing Matrigel^TM^ and a high concentration of rCOL in serum-free medium, Table S4: Hydrogel construct containing Matrigel^TM^/rCOL, and SeaPlaque^TM^ agarose in serum-free medium, Table S5: Hydrogel construct containing hCOL and SeaPlaque^TM^ agarose in serum-free medium.
