# Supplementary material for: Characterization of Tissue Engineered Endothelial Cell Networks in Composite Collagen-Agarose Hydrogels
Source: Gels. 2020 Sep 3;6(3):27. doi: 10.3390/gels6030027 (PMC7559300; doi:10.3390/gels6030027)
Supplement: Supplementary file 1 [file gels-06-00027-s001.pdf]

Supplementary Materials for

Characterization of tissue engineered endothelial networks in composite collagen-agarose hydrogels

Houda Ichanti, Sanja Sladic, Stefan Kalies, Axel Haverich, Birgit Andrée * and Andres Hilfiker *

Table S1. Control hydrogel construct containing Matrigel™/rCOL in serum-free medium.

Substance	Stock concentration	Volume per construct (µL)	Final concentration
rCOL	5 mg/mL	58	1 mg/mL
Ampuwa water	-	14	-
Gel medium	-	74	-
NaOH	0.4 mM	12.5	-
Matrigel™	8-10 mg/mL	31	0.8-1mg/mL
Cell suspension in SFM	-	185.5	-

Table S2. Control hydrogel construct containing hCOL in serum-free medium.

Substance	Stock concentration	Volume per construct (µL)	Final concentration
hCOL	3 mg/mL	190	1.9 mg/mL
Ampuwa water	-	14	-
SF gel medium	-	74	-
NaOH	0.4 mM	20	-
Cell suspension in SFM	-	77	-

Table S1: Hydrogel construct containing Matrigel™ and a high concentration of rCOL in serum-free medium.

Substance	Stock concentration	Volume per construct (µL)	Final concentration
rCOL	8.34 mg/mL	109	3 mg/mL
Ampuwa water	-	14	-
SF gel medium	-	74	-
NaOH	0.4 mM	23.5	-
Matrigel™	8-10 mg/mL	31	0.8-1 mg/mL
Cell suspension in SFM	-	52	-

Table S2: Hydrogel construct containing Matrigel™/rCOL, and SeaPlaque™ agarose in serum-free medium.

Substance	Stock concentration	Volume per construct (µL)	Final concentration
rCOL I	5 mg/mL	58	1 mg/mL
Ampuwa water	-	14	-
SF gel medium	-	74	-
NaOH	0.4 mM	12.5	-
Matrigel™	8-10 mg/mL	31	0.8-1 mg/mL
Cell suspension in SFM	-	128.5	-

SeaPlaque™ Agarose	0.4%	57	0.03 %

Table S3: Hydrogel construct containing hCOL and SeaPlaque™ agarose in serum-free medium.

Substance	Stock concentration	Volume per construct (μL)	Final protein concentration
hCOL	5 mg/mL	190	1 mg/mL
Ampuwa water	-	14	-
SF gel medium	-	74	-
NaOH	0.4 mM	20	-
Cell suspension in SFM	-	20	-
SeaPlaque™ Agarose	0.4%	57	0.03 %
